# Development of a stable ERroGFP variant suitable for monitoring redox dynamics in the ER

**DOI:** 10.1042/BSR20160027

**Published:** 2016-04-15

**Authors:** Jun Hoseki, Asami Oishi, Takaaki Fujimura, Yasuyoshi Sakai

**Affiliations:** * Research Unit for Physiological Chemistry, the Center for the Promotion of Interdisciplinary Education and Research, Kyoto University, Kitashirakawa-oiwake-cho, Sakyo-ku, Kyoto 606-8502, Japan; † Division of Applied Life Sciences, Graduate School of Agriculture, Kyoto University, Kitashirakawa-oiwake-cho, Sakyo-ku, Kyoto 606-8502, Japan

**Keywords:** endoplasmic reticulum (ER) redox state, glutathione, protein quality control, redox probe

## Abstract

The endoplasmic reticulum (ER) is an essential organelle for cellular metabolic homeostasis including folding and maturation of secretory and membrane proteins. Disruption of ER proteostasis has been implicated in the pathogenesis of various diseases such as diabetes and neurodegenerative diseases. The ER redox state, which is an oxidative environment suitable for disulfide-bond formation, is essential for ER protein quality control. Hence, detection of the ER redox state, especially in living cells, is essential to understand the mechanism by which the redox state of the ER is maintained. However, methods to detect the redox state of the ER have not been well-established because of inefficient folding and stability of roGFP variants with oxidative redox potential like roGFP-iL. Here we have improved the folding efficiency of ER-targeted roGFP-iL (ERroGFP-iL) in cells by introducing superfolder GFP (sfGFP) mutations. Four specific amino acid substitutions (S30R, Y39N, T105N and I171V) greatly improved folding efficiency in *Escherichia coli* and in the ER of HeLa cells, as well as the thermostability of the purified proteins. Introduction of these mutations also enhanced the dynamic range for redox change both *in vitro* and in the ER of living cells. ER-targeted roGFP-S4 (ERroGFP-S4) possessing these four mutations could detect physiological redox changes within the ER. ERroGFP-S4 is therefore a novel probe suitable for monitoring redox change in the ER. ERroGFP-S4 can be applied to detect aberrant ER redox states associated with various pathological conditions and to identify the mechanisms used to maintain the redox state of the ER.

## Abstract (precis)

We have created ERroGFP-S4, a novel ER redox probe suitable for monitoring redox dynamics in the oxidative environment of the ER. ERroGFP-S4 can be used for detection of aberrant ER redox states related to various physiological and pathological conditions.

## INTRODUCTION

The endoplasmic reticulum (ER) is an organelle that is essential for cellular metabolic homoeostasis; the ER plays a central role in homoeostasis of secretory and membrane proteins as well as homoeostasis of calcium and lipids. The ER handles the folding and maturation of massive amounts of proteins, consisting of one third of the total cellular proteins, and therefore the ER has developed a sophisticated protein quality control system [1]. When cellular stresses that perturb ER proteostasis cause the abnormal accumulation of misfolded and/or unfolded proteins in the ER over the quality control capacity (such a condition is called ER stress), the unfolded protein response (UPR) is induced to restore ER proteostasis [2]. Chronic ER stress, which leads to apoptosis, has been implicated in the pathogenesis of many diseases such as diabetes and neurodegenerative diseases [3].

The ER redox state is essential for protein quality control in the ER. Disulfide-bond formation is required for structural formation and functions of many proteins that are folded and maturated in the ER; therefore, compared with the cytosol, the ER maintains an oxidative environment suitable for disulfide-bond formation [4]. In addition to the oxidative environment of the ER, ER-localized protein disulfide isomerase (PDI) family proteins, composed of tandem thioredoxin-like domains, play thiol management roles in ER protein quality control [5]: PDI and ERp57 promote oxidative folding by introducing the correct disulfide bonds and ERdj5 facilitates ER-associated degradation of terminally misfolded proteins by cleaving their disulfide bonds [6–8].

Glutathione, which is synthesized in the cytosol, has been considered to be the major redox molecule that defines the ER redox state [9,10]. In yeast, overexpression of *GSH1* (γ-glutamylcysteine synthetase, the rate-limiting enzyme of glutathione biosynthesis) resulted in accumulation of misfolded proteins in the ER and induced the UPR, whereas knockout of *GSH1* rescued the growth defect of a temperature sensitive *ERO1* (an ER-resident oxidase essential for oxidative folding of proteins via PDI) mutant at high temperature [11]. In mammalian cells, cytosolic glutathione was also reported to maintain the ER redox state by counterbalancing against Ero1L (Ero1-like protein) [12]. In addition to maintenance of the oxidative ER redox state, glutathione is necessary for reduction of PDI and ERp57 [13]. Therefore, maintenance of the glutathione redox state in the ER is assumed to contribute to ER protein quality control. Recently, however, Tsunoda et al. [14] reported that degradation of glutathione in the ER did not affect the maturation of the LDL receptor and the degradation of misfolded α1-antitrypsin. Therefore the necessity of glutathione in ER protein quality control is still controversial. In addition, the mechanism of glutathione transport across the ER membrane, by which the ER redox state should be regulated and maintained, is still unclear.

Biochemical quantification of GSSG and GSH in the microsome fraction was performed to estimate the ER redox state as the GSH/GSSG ratio in the fraction [4,15]. However, fractionation of microsomes and spontaneous oxidation during the preparation of the microsome fraction hamper accurate determination of GSSG and GSH content. In addition, the glutathione redox state is defined as [GSH]^2^/[GSSG] because one molecule of GSSG is equilibrated with two molecules of GSH. Therefore, the concentrations of GSH and GSSG, but not their contents are essential for determining the glutathione redox state. However, accurate estimation of ER volume is technically difficult.

About a decade ago, roGFP, which harbours two cysteines located on neighbouring β-strands, was developed for real-time detection of the intracellular glutathione redox state [16,17]. roGFP has two excitation peaks derived from the neutral and the anion forms of the chromophore, and the equilibrium between the two forms is redox-dependent. Under oxidizing conditions in which the disulfide bond between the redox-sensitive cysteines, Cys-147 and Cys204 is formed, the population of the neutral form is increased at the expense of the anion form. When the disulfide bond is cleaved under reducing conditions, the population of the neutral form is decreased and that of the anion form is increased. Thus, roGFP is a reporter of the redox-dependent ratiometric fluorescence property. However, the initial versions of roGFP, roGFP1 and roGFP2, had reduced redox potentials inappropriate for detection of redox change, especially oxidative change, under the oxidative redox environment of the ER. This is because the oxidized form of these roGFPs was thermodynamically stable under oxidative redox conditions. ER-targeted roGFP2 (EroGFP) in *Saccharomyces cerevisiae* detected only reductive changes in the ER redox state following ER stress induced by tunicamycin [18]. roGFP1 was modified by inserting an amino acid residue at the position next to Cys147, one of the cysteines that forms the intramolecular disulfide bond [19]. Owing to the decrease in the thermodynamic stability of the disulfide bond by this insertion, roGFP-iX variants (iX: X (an amino-acid residue) is inserted) had oxidized redox potentials suitable for measurement of the ER redox state. However, ER-targeted roGFP-iE and roGFP-iL showed weak fluorescence in the ER in living cells due to low folding efficiency [20]. Avezov et al. [21] detected the ER redox state using lifetime imaging instead of fluorescence intensity, and van Lith et al. [22] monitored real-time changes in the ER redox state by ER-targeting roGFP-iL with strong excitation light, although in the yeast *Pichia pastoris,* Delic et al. [23] measured the ER redox state by using roGFP-iL and -iE.

We attempted to monitor the ER redox state by using ER-targeted roGFP-iL (ERroGFP-iL) in HeLa cells. However, the fluorescence intensity of ERroGFP-iL was not sufficient for measurement of the ER redox state due to its low folding efficiency in the ER. In the present study, we have improved the folding efficiency of roGFP-iL by introducing mutations corresponding to those in superfolder GFP (sfGFP) [24]. Our newly created roGFP-iL mutants have greatly increased folding efficiency in *Escherichia coli* and even in the ER environment. Among these mutant forms, ERroGFP-S4 could detect real-time and physiological redox dynamics in the ER and therefore is suitable for monitoring redox changes in the ER in living cells.

## EXPERIMENTAL

### Cell culture, construction of plasmids, transfection and preparation of stable cells

HeLa cells were grown in Dulbecco's modified Eagle medium with 10% fetal bovine serum and antibiotics (100 units/ml penicillin and 100 μg/ml streptomycin) under humidified air containing 5% CO_2_ at 37°C. Plasmids expressing roGFP-iL in *E. coli* (pQE30-ro-iL) and roGFP1 in mammalian cells (pEGFP-N1-ro1) were kindly provided by Dr S. James Remington (Oregon University). The ER-targeted roGFP-iL coding sequence in which leucine was inserted after Cys147 and the following six mutations (F64L, F99S, H148S, M153T, V163A and I167T) were added into the roGFP1 sequence in pEGFP-N1-ro1 was artificially synthesized with the signal sequence of mouse calreticulin in the N-terminus and KDEL sequence in the C-terminus and subcloned into pIRES-puro3 (Clontech) with *NheI* and *NotI* sites. Additional mutations (S30R, T39N, N105T, I171V, Y145F and A206V) derived from sfGFP were generated using a QuikChange Lightning Multi Site-Directed Mutagenesis Kit (Agilent Tech.). Construction of mammalian expression plasmids (pCAGGS) encoding the mouse Ins2 C96Y mutant was described previously [25]. Cells were transfected with plasmids using Lipofectamine 2000 (Invitrogen). For preparation of stable cells expressing ER-targeted roGFP-S4, HeLa cells were transfected with pIRES-puro3-ERroGFP-S4 and selected with 1 μg/ml of puromycin.

### Antibodies

Anti-GFP JL-8 mouse monoclonal (Clontech) and anti-β-actin (Sigma–Aldrich) antibodies were used for immunoblotting and anti-Hsp47 SPA-470 mouse monoclonal (Enzo Life Sciences) and anti-GFP rabbit polyclonal (Life Technology) antibodies for immunostaining.

### Protein expression in *E. coli* and purification

*E. coli* BL21(DE3) cells (Novagen) carrying 6xHis-tagged roGFP-iLs expression plasmids were grown at 37°C until the OD_600_ reached 0.5–0.7, at which point expression of the recombinant proteins was induced at 25°C (at 25°C and 37°C for expression check) for 16 h by adding 0.4 mM isopropyl-β-D-thiogalactoside. As an expression check, harvested cells expressing roGFP-iLs were lysed by sonication in a lysis buffer (50 mM NaH_2_PO_4_ (pH 8.0 adjusted using NaOH) containing 300 mM NaCl and 10 mM imidazole) and then the cell lysate was subjected to centrifugation (12000 × ***g*** for 10 min at 4°C). For purification of His-tagged recombinant roGFP-iLs, harvested cells were resuspended in the lysis buffer and lysed by using a French press and the supernatant of the cell lysate was affinity-purified at room temperature using Ni-nitrilotriacetic acid resin (Qiagen) according to the manufacturer's instructions. The his-tagged roGFP-iLs were eluted with lysis buffer containing 250 mM imidazole, and exchanged into assay buffer (50 mM potassium phosphate buffer (pH 7.0) containing 150 mM KCl and 1 mM EDTA) using PD-10 (GE Healthcare).

### Thermostability of purified roGFP-iLs

Purified roGFP-iLs were incubated at high temperatures (75, 80, 85 and 95°C) and then separated into supernatant and pellet by centrifugation at 15000 × ***g*** for 5 min at 4°C. The resultant supernatant and pellet were resolved by SDS-PAGE and followed by CBB staining.

### Redox titration of purified roGFP-iLs with glutathione by the fluorescence ratio

Purified roGFP-iLs (1 μM) was incubated at 30°C for 16 h in a degassed assay buffer with different GSSG and GSH concentrations in which the total glutathione concentration was 2 mM. After incubation, excitation spectra (emission at 510 nm) of roGFP-iLs were measured on a Shimadzu RF-5300PC fluorescence spectrophotometer. The ratio of the fluorescence intensity (F ratio) at 510 nm (Ex390/Ex465 for iL, S4, and S5-6 and Ex387/Ex484 for S5-4 and S6) was plotted against [GSH]^2^/[GSSG]. The redox equilibrium constant of roGFP-iLs with glutathione (*K*_eq_) for the following reaction was expressed as follows:

$$\mathrm{roGF}P_{\mathrm{red}}+\mathrm{GSSG}\;\overset{K_{\mathrm{eq}}}{↔}\;\mathrm{roGF}P_{\mathrm{oxi}}+2\mathrm{GSH}$$

$$\;K_{\mathrm{eq}}=\frac{\;\left[\mathrm{roGF}P_{\mathrm{oxi}}\right]{\left[\mathrm{GSH}\right]}^{2}}{\left[\mathrm{roGF}P_{\mathrm{red}}\right]\left[\mathrm{GSSG}\right]}\;$$

*K*_eq_, dynamic range ((F ratio_oxi_ - F ratio_red_)/F ratio_red_) and F ratio_red_ (the lowest ratio) were calculated by non-linear fitting measured F ratios to the following equation using KaleidaGraph 4 (HULINKS):

$$F\;\mathrm{ratio}\;=\;\mathrm{dynamic}\;\mathrm{range}\times \frac{\frac{\;{\left[\mathrm{GSH}\right]}^{2}}{\left[\mathrm{GSSG}\right]}}{\frac{\;{\left[\mathrm{GSH}\right]}^{2}}{\left[\mathrm{GSSG}\right]}+K_{\mathrm{eq}}}+F\;\mathrm{rati}o_{\mathrm{red}}$$

### Confocal fluorescence and immunofluorescence microscopy of the fixed cells

Cells overexpressing ERroGFP-iLs were fixed with 4% paraformaldehyde for 20 min at room temperature, washed with phosphate buffered saline without calcium and magnesium (PBS (-)) containing 0.2% Triton X-100, and incubated and permeabilized with PBS (-) containing 1% glycerol, 1% bovine serum albumin, 1% normal goat serum and 0.2% Triton X-100 for 1 h. The fixed and permeabilized cells were incubated with rabbit anti-GFP antibody for 2 h, mouse anti-Hsp47 for 1 h, and then with Alexa Fluor 546-conjugated goat anti-rabbit and Alexa Fluor 633-conjugated goat anti-mouse (Life Technology) as the secondary antibodies for 1 h. ERroGFP-iLs in the fixed cells were excited using a 488 nm laser and fluorescence was detected using a 500–530 nm filter. Multicolour confocal fluorescence images were obtained by sequential scanning using an LSM 510 META confocal microscope (Carl Zeiss).

### Sample preparation for Western blotting

Cells overexpressing ER-targeted roGFP-iL mutants were washed with PBS (-) and lysed with 50 mM HEPES–NaOH buffer (pH 7.4) containing 150 mM NaCl and 1% Nonidet P-40, freshly supplemented with protease inhibitor cocktail (Nacalai Tesque) on ice for 20 min. The cell lysate was centrifuged at 12000 × ***g*** for 20 min at 4°C. The supernatant was collected and the pellet was resuspended by sonication in the above-mentioned NP-40 lysis buffer for Western blotting.

### Monitoring and measurement of fluorescence intensities and ratios for ER-targeted roGFP-iL mutants

Fluorescence images of HeLa cells expressing ER-targeted roGFP-iL mutants were acquired at 37°C using an LSM 510 META confocal microscope. The cells expressing ER-targeted roGFP-iL mutants were excited using 405 nm and 458 nm lasers and their fluorescence was detected using a 500–530 nm filter. ImageJ was used for the following quantification of the acquired images. For quantification of fluorescence intensity, 15 cells from a minimum of five fields were randomly selected for each measurement. The fluorescence intensity within a particular size of circle was quantified (three circles per cell) and the background intensity within the same size of circle was subtracted. The fluorescence intensity ratio (Ex405/Ex458), which indicates the ER redox state, was calculated using the quantified fluorescence intensities.

## RESULTS

### Fluorescence intensity and folding efficiency of ERroGFP-iL in the ER

**Figure 1:**
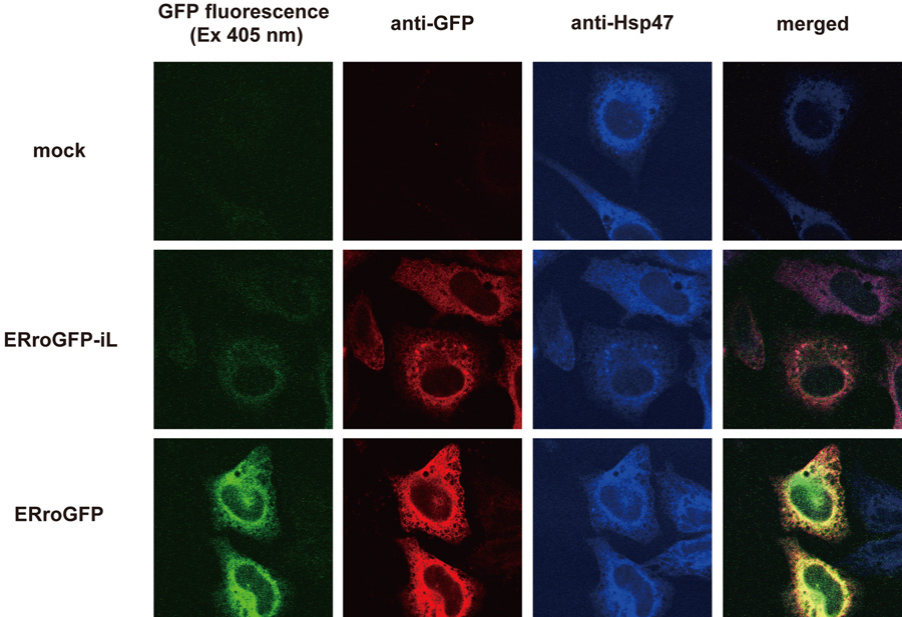
Fluorescence and immunofluorescence imaging of ER-targeted roGFP and roGFP-iL Fluorescence images of HeLa cells transfected with ER-targeted roGFP and roGFP-iL were acquired (green) after fixation with 4% PFA and the cells were also co-stained with anti-GFP (red) and anti-Hsp47 (blue, ER marker).

HeLa cells were transfected with roGFP-iL targeted to the ER, and fluorescence intensity and intracellular localization of ERroGFP-iL were observed under confocal microscopy. The fluorescence intensity of ERroGFP-iL was very weak compared to that of ERroGFP. However, although the immunofluorescence signal of ERroGFP-iL was less than that of ERroGFP, it was at a detectable level, and it co-localized with the ER marker Hsp-47 (Figure 1). These data suggest that the folding efficiency of ERroGFP-iL in the ER is much lower than that of ERroGFP.

### Construction of roGFP-iL mutants, their folding efficiency in *E. coli* and their thermostability *in vitro*

**Figure 2:**
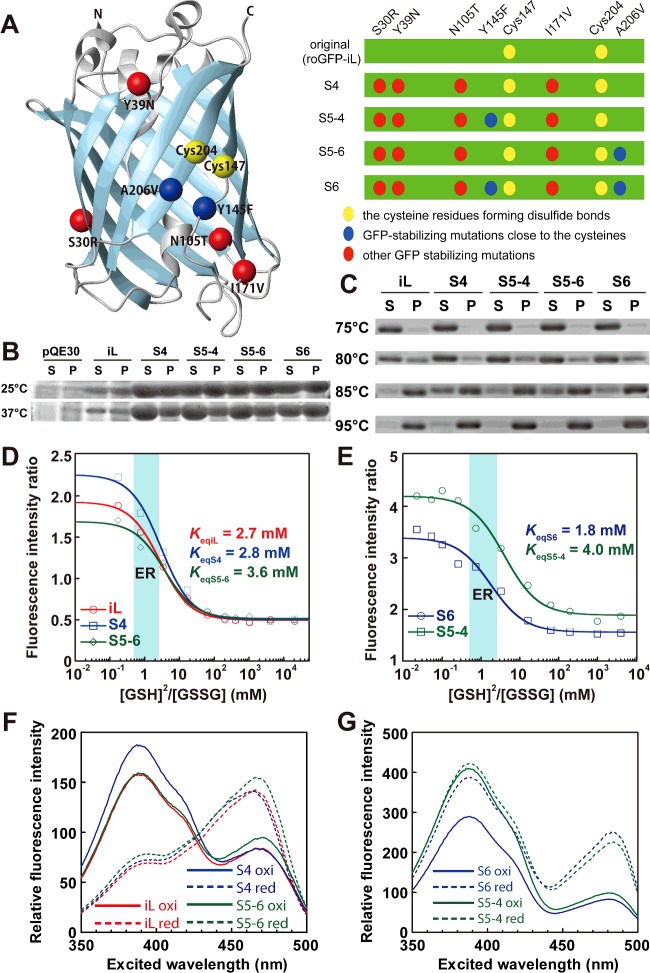
Biochemical properties of purified roGFP-iL mutants (**A**) The Cα atoms of residues mutated in the roGFP-iL mutants are shown as spheres in the ribbon structure of roGFP drawn by Molmol software (left). Schematic representation of roGFP-iL mutants (right). (**B**) Expression of roGFP-iL mutants in *E. coli*. After disruption of cells expressing roGFP-iL mutants, the cell lysate was fractionated by centrifugation. SDS-PAGE of the resultant supernatant (S) and pellet (P) fractions was performed and the gel was CBB-stained. (**C**) Thermostability of the recombinant roGFP-iLs. Purified roGFP-iLs were incubated at the indicated temperature and after centrifugation supernatant and pellet were resolved by SDS-PAGE. (**D** and **E**) Redox titration of roGFP-iL, S4 and S5-6 (D) and S5-4 and S6 (E) with glutathione. The redox equilibrium constant (*K*_eq_) with glutathione and the dynamic range for glutathione redox change were determined by non-linear fitting the data according to the following equation: 
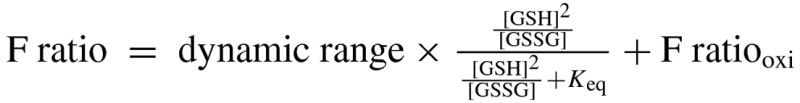
, where F ratio indicates the ratio of fluorescence intensity and the dynamic range is (F ratio_oxi_ - F ratio_red_)/F ratio_red_. (**F** and **G**) Excitation spectra (emission at 510 nm) of roGFP-iL, S4 and S5-6 (F) and S6 and S5-4 (G). Oxi indicates the most oxidizing condition in the glutathione titration and red indicates the most reducing condition in the glutathione titration.

To enhance the folding efficiency and fluorescence intensity of roGFP-iL in the ER of living cells, mutations derived from sfGFP [24] were introduced into roGFP-iL. sfGFP has six mutations (S30R, Y39N, N105T, Y145F, I171V and A206V) and can fold efficiently, even when it is fused to poorly folded proteins. Four mutations (S30R, Y39N, N105T and I171V) were introduced into roGFP-iL, which was termed S4 (Figure 2A). The other mutations, Y145F and A206V, are adjacent to the two disulfide bond-forming cysteine residues (Cys147 and Cys204) of roGFP-iL, and thus might influence the redox potential of roGFP-iL. Therefore, mutants with either or both of the two mutations in S4 were also constructed and termed S5-4, S5-6 and S6 respectively (Figure 2A).

roGFP-iL and its four mutants were expressed in *E. coli* BL21(DE3) cells at 25°C and 37°C for 18 h. The expression levels of all four mutants were dramatically increased compared to that of the original roGFP-iL at both temperatures (Figure 2B). Furthermore, the soluble fraction of S4 expressed at 25°C was considerably larger than the insoluble fraction. In contrast, the soluble fractions of roGFP-iL and the other mutants were almost the same or less than the corresponding inclusion-body fractions. The soluble fractions of S4, S5-4 and S5-6 expressed at 37°C were considerably larger than the corresponding inclusion-body fractions, whereas the soluble fractions of roGFP-iL and S6 were almost the same or less than the corresponding insoluble fractions.

Next, the thermostability of purified roGFP-iL and its mutants was examined after incubation for 10 min at high temperatures. All of the roGFP-iLs were clearly soluble at 75°C and substantially soluble even at 80°C (Figure 2C). The ratios of soluble to insoluble fractions of S4 and S5-6 at 80°C were slightly higher than those of roGFP-iL, S5-4 and S6. Substantial amounts of S4 and S5-6 were still soluble at 85°C; furthermore, a small amount of soluble S4 was observed even at 95°C, although the others were almost insoluble.

These results suggest that introduction of the mutations into roGFP-iL remarkably enhanced the expression level, especially for S4 and S5-6, and greatly improved the folding efficiency (ratio of soluble to insoluble) and thermostability compared to roGFP-iL.

### Fluorescence properties of ERroGFP-iL mutants

The fluorescence spectra of the recombinant roGFP-iLs purified from *E. coli* cells were determined. Emission spectra following excitation of S4 and S5-6 at 458 nm showed a single peak around 500 nm, which was almost the same as that of roGFP-iL (see Supplementary Figure S1). The peaks of the emission spectra of S6 and S5-4 were slightly red-shifted to 505 nm. The excitation spectra of iL, S4 and S5-6 showed two peaks at 390 nm and 465 nm (see Supplementary Figure S2). S6 and S5-4 also showed two peaks in the excitation spectra but the latter wavelength peak was red-shifted to 485 nm. When oxidized by the oxidant 4,4’-dipyridyl sulfide (DPS), S4 and S5-6 showed higher fluorescence intensities for the 390 nm peak and lower intensities for the 465 nm peak, whereas when reduced by DTT showed lower intensities for the former peak and higher intensities for the latter peak, similar to roGFP-iL. These data indicate that S4 and S5-6 maintain the redox-dependent ratiometric properties of the original roGFP-iL. In contrast, S6 and S5-4 showed lower fluorescence intensities for both peaks when oxidized than when reduced. Therefore, the redox-dependent ratiometric properties of S6 and S5-4 were diminished (see Supplementary Figure S2).

Next, the fluorescence ratio (Ex390/Ex465 or Ex387/Ex484) of roGFP-iLs was titrated with glutathione. Redox equilibrium constants for all of the mutants with glutathione were almost the same as that of roGFP-iL, which is suitable for measuring an oxidizing ER redox state (Figures 2D and 2E). This result suggests that introduction of the mutations derived from sfGFP, even the two mutations (Y145F and A206V) adjacent to the disulfide-bonded cysteines, did not influence the redox potential of the original roGFP-iL. S4 substantially increased the fluorescence intensity of only the first peak under oxidized conditions compared to roGFP-iL, showed similar fluorescence intensity at the second peak in oxidized conditions, and at both peaks under reduced conditions compared to roGFP-iL, resulting in an increase in the fluorescence ratio (Ex390/Ex465) of S4 (Figure 2F). S5-6 slightly increased the fluorescence intensity only at the second peak under both conditions. Therefore, S4 exhibited a 20% higher dynamic range of fluorescence ratio (Ex390/Ex465) for glutathione redox change, whereas S5-6 had approximately a 15% lower dynamic range compared to roGFP-iL (Table 1). S6 had decreased fluorescence intensity at both peaks when oxidized and increased intensity when reduced (Figure 2G). For S5-4, the fluorescence intensity of only the second peak was decreased when oxidized. Consequently the dynamic range of the fluorescence ratio (Ex387/Ex484) of S6 and S5-4 in glutathione redox change was considerably diminished to half that of roGFP-iL (Table 1). These results suggest that introduction of the four common mutations among the mutants increased the dynamic range for glutathione redox change, whereas introduction of the Y145F mutation into S6 and S5-4 significantly increased the overall fluorescence intensity, but attenuated the dynamic range by eliminating the redox-dependent ratiometric property. The A206V mutation in S5-6 and S6 also decreased the ratiometric property.

**Table 1:**
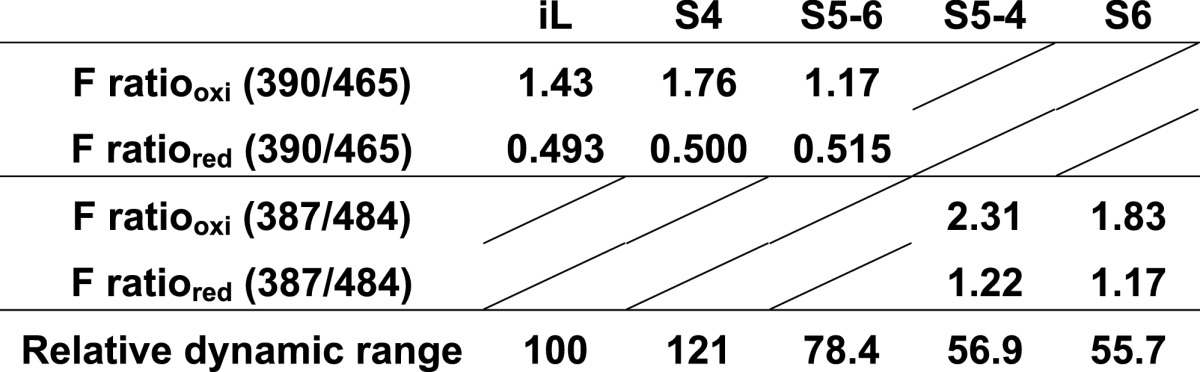
Dynamic range of the purified roGFP-iLs Here the fluorescence ratio of the reduced form (F ratio_red_) was calculated using the dynamic range and F ratio_oxi_ was derived from the non-linear fitted sigmoid curves (see Figures 2D and 2E). The dynamic range relative to that of iL is also shown.

### Fluorescence intensity and folding efficiency of roGFP-iL mutants in the ER

**Figure 3:**
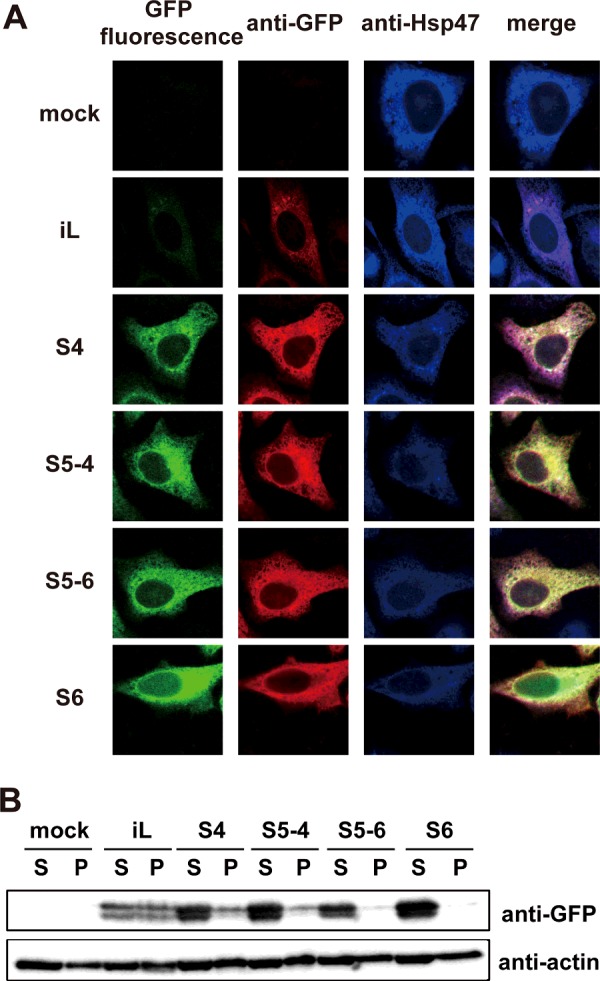
Fluorescence properties of ER-targeted roGFP-iL mutants in the ER (**A**) Fluorescence images of HeLa cells transfected with ER-targeted roGFP-iL and its mutants were acquired (green) after fixation with 4% PFA. The cells were also co-stained with anti-GFP (red) and anti-Hsp47 (blue, ER marker). (**B**) Immunoblotting of ER-targeted roGFP-iL and its mutants with anti-GFP in 1% NP-40-soluble (S) and -insoluble (P) cell fractions.

The mutations derived from sfGFP dramatically improved the folding efficiency in *E. coli*, although the Y145F mutation abolished the redox-dependent ratiometric property of roGFP-iL in the purified recombinant proteins. To confirm the effectiveness of the mutations introduced into roGFP-iL on the folding efficiency in the ER environment, ER-targeted roGFP-iLs were transiently transfected into HeLa cells. Substantial fluorescence (excited at 488 nm) of the roGFP-iL mutants was observed, in contrast with weak fluorescence of roGFP-iL (Figure 3A). The fluorescence intensities of S6 and S5-4 were higher than those of S4 and S5-6, which is consistent with the overall higher fluorescence intensity of the purified S6 and S5-4 (Figure 2F). Immunofluorescence using a GFP antibody showed higher expression of the mutants compared to the original roGFP-iL and the roGFP-iL mutants co-localized with an ER marker Hsp47 (Figure 3A).

Next, the protein levels of these ER-targeted roGFP-iLs were examined 24 h after transfection by Western blotting. The expression levels of the mutants were significantly increased compared to that of the original roGFP-iL, although a slightly smaller band was seen for all of the roGFP-iLs in addition to the full-length band (Figure 3B). Almost all of the mutant proteins were detected in the NP-40 soluble fraction; in contrast about half of the roGFP-iL was detected in the NP-40 insoluble fraction. These data suggest that introduction of mutations into roGFP-iL improved the folding efficiency of roGFP-iL in the ER environment as well as in *E. coli*. Based on the fluorescence, all of the ER-targeted roGFP-iL mutants were expressed well in the ER at levels sufficient for following redox dynamics in the ER.

### Redox-dependent fluorescence properties of the roGFP-iL mutants in the ER

**Figure 4:**
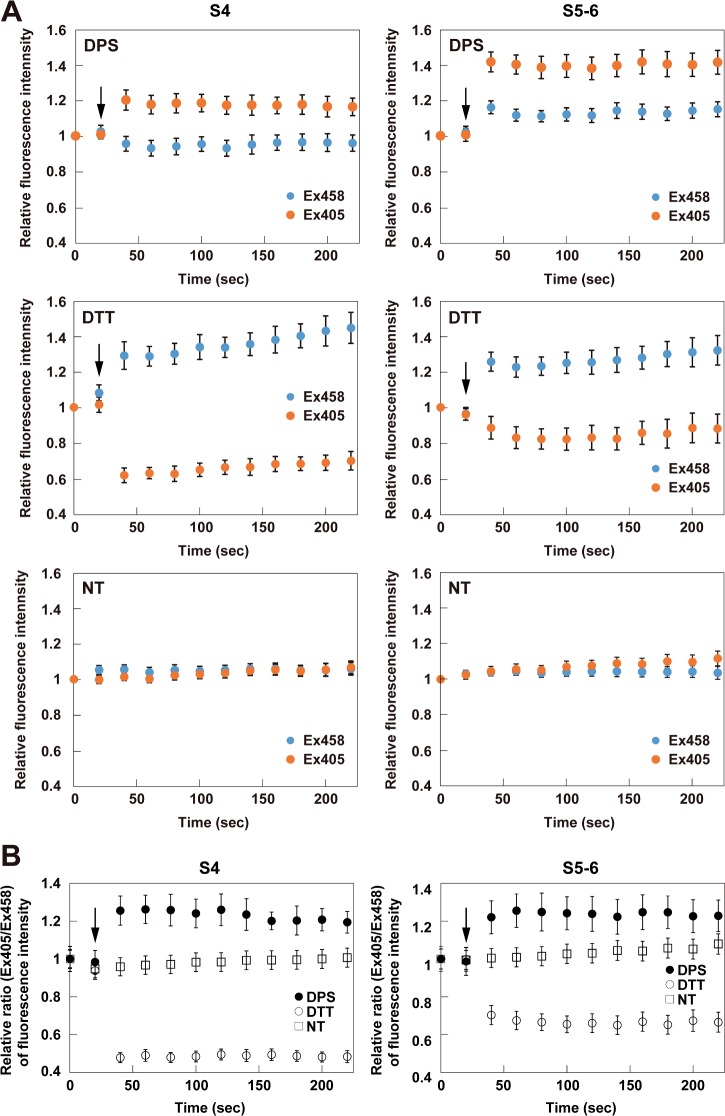
Fluorescence properties of ER-targeted roGFP-iL, S4 and S5-6 in the ER (**A** and **B**) Time lapse of relative fluorescence intensities (Ex405 and Ex458) (A) compared to those at time 0 and relative fluorescence ratios (Ex405/Ex458) (B) compared to those at time 0 of ERroGFP-iL and its mutants, S4 and S5-6. ER-targeted roGFP-iLs were transiently expressed in HeLa cells and then oxidoreductants (final 0.5 mM DPS or 5 mM DTT) were added 20 s after the start of the measurement (arrow indicates the addition). The fluorescence images were scanned every 20 s until 220 s. The measured relative fluorescence intensities are shown as the mean ± S.E.M. of three independent measurements.

**Figure 5:**
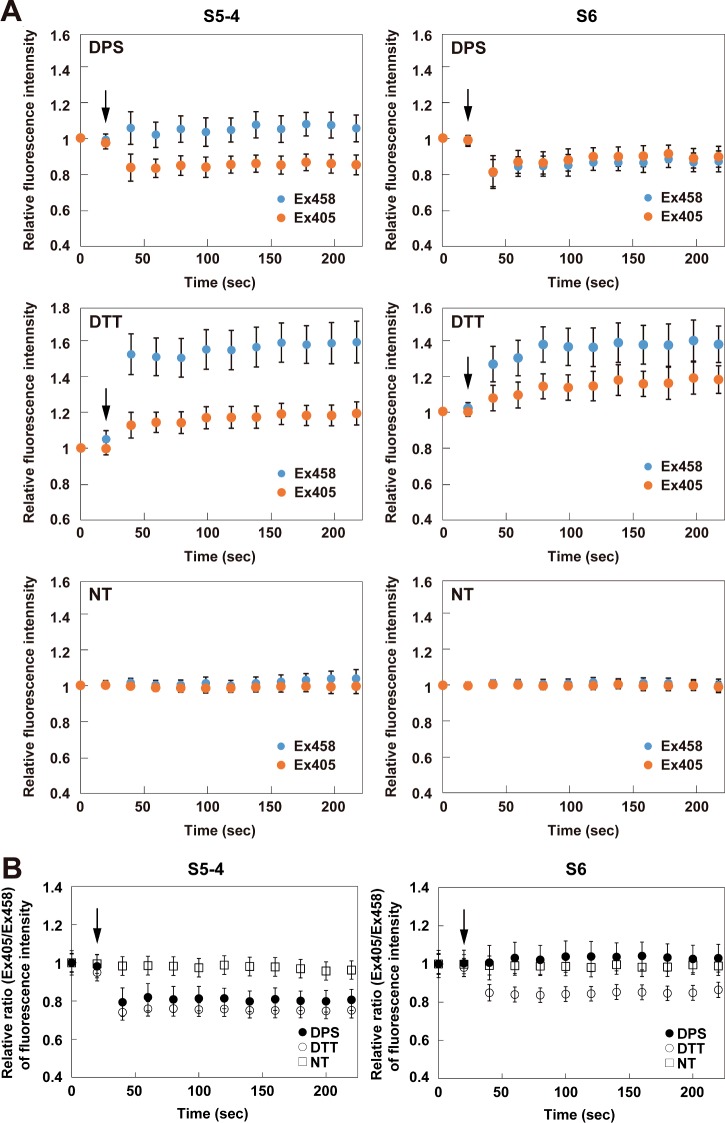
Fluorescence properties of ER-targeted S5-4 and S6 in the ER (**A** and **B**) Time lapse of relative fluorescence intensities (Ex405 and Ex458) (A) compared to those at time 0 and relative fluorescence ratios (Ex405/Ex458) (B) compared to those at time 0 of the ERroGFP-iL mutants, S5-4 and S6. ER-targeted roGFP-iLs were transiently expressed in HeLa cells and then oxidoreductants (final 0.5 mM DPS or 5 mM DTT) were added 20 s after the start of the measurement (arrow indicates the addition). The fluorescence images were scanned every 20 s until 220 s. The measured relative fluorescence intensities are shown as the mean ± S.E.M. of three independent measurements.

In order to investigate the redox-dependent fluorescence properties of the roGFP-iL mutants in the ER, their fluorescence intensities excited at 405 nm and 458 nm after addition of DPS (oxidant) or DTT (reductant) were monitored. After DPS addition, the fluorescence intensity of S4 was immediately increased when excited at 405 nm and slightly decreased when excited at 458 nm as shown in Figure 4A, resulting in a significant increase in the fluorescence intensity ratio (Ex405/Ex458) compared to the non-treatment condition (Figure 4B). In contrast, the fluorescence of S4 after DTT addition promptly decreased when excited at 405 nm and increased when excited at 458 nm, resulting in a significant decrease of the ratio (Figures 4A and 4B). Therefore, S4 demonstrated redox-dependent ratiometric properties in the ER of living cells. S5-6 similarly showed redox-dependent ratiometric properties; however, the dynamic range of S5-6 between the oxidized and reduced conditions was narrower compared to that of S4 because the fluorescence ratio of S5-6 when reduced was higher than that of S4 (Figure 4B and Table 2). The dynamic ranges of S6 and S5-4 were markedly attenuated in contrast with those of S4 and S5-6, because the fluorescence ratio of the oxidized proteins was unchanged in S6 or decreased in S5-4, whereas those of the reduced proteins were decreased in both mutants (Figure 5 and Table 2). All of the mutants retained constant fluorescence intensities when excited at both wavelengths and showed approximately constant fluorescence ratios under non-treatment conditions (Figures 4A and 5A, lower panels).

**Table 2:**
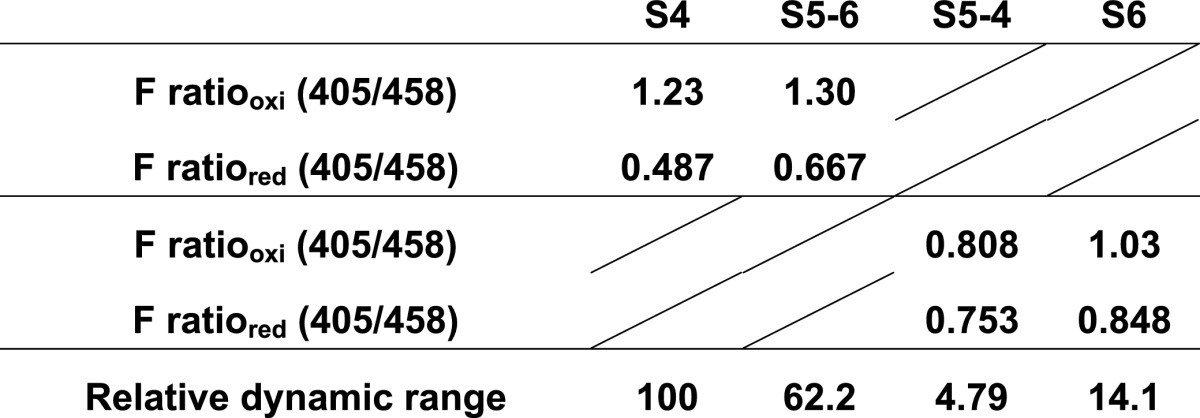
Dynamic range of ERroGFP-iLs in the ER Here the dynamic range relative to that of S4 is shown, where the dynamic range is (F ratio_red_ - F ratio_oxi_)/F ratio_oxi_.

Taken together, S4 and S5-6 showed useful redox-dependent ratiometric properties for detecting the dynamics of the ER redox state. These results are consistent with the *in vitro* results of the fluorescence measurements (Figures 2D and 2E and Table 1). Among the mutants, S4 had the broadest dynamic range for redox change in the ER, and therefore is the most suitable for detection of the ER redox state.

### S4 was capable of detecting physiological redox changes in the ER

To detect physiological redox dynamics in the ER, we have established HeLa cells that stably express S4. The redox change in the ER following overexpression of murine Ins2 mutant (C96Y) was detected by the S4 expressing cells. The C96Y mutation of mIns2 exposes its hydrophobic surface due to the absence of the A7–B7 disulfide bond and as a result, the Ins2 mutant tends to aggregate [26]. In a mouse line with the mutation (called Akita mouse), the Ins2 mutant protein is misfolded and accumulates in the ER of pancreatic β-cells, which leads to death of β-cells through ER stress, and eventually causes diabetes. Therefore, the Akita mouse has been considered to be a mouse model for type II diabetes [27].

**Figure 6:**
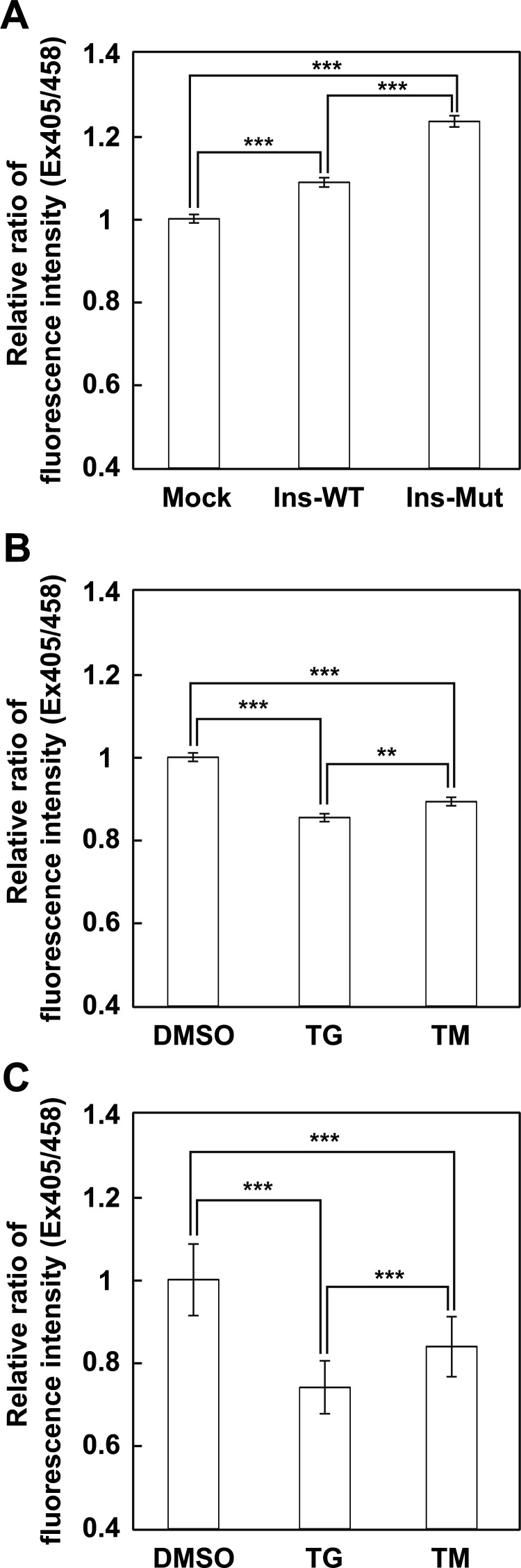
S4 detected physiological redox changes in the ER (**A**) Relative fluorescence intensity ratio 48 h after transfection with Ins2 (WT and C96Y mutant) in cells stably expressing S4. Relative fluorescence intensity ratio at 8 h (**B**) and at 16 h (**C**) after treatment of the ER with stress inducers. The quantified relative fluorescence ratios are shown as the mean ± S.E.M. of three independent measurements. **: *P*<0.01, ***: *P*<0.001

Overexpression of the Ins2 C96Y mutant, which is misfolded and accumulated in the ER, considerably increased the fluorescence ratio (Ex405/Ex458), indicating oxidation of the ER redox state, at 48 h after the transfection (Figure 6A). The Ins2 WT, which is correctly folded and secreted out of the cells, a slight increase in the fluorescence ratio was also observed compared to mock cells, indicating an oxidized ER redox state.

Finally, ER redox changes under ER stress conditions induced by tunicamycin, a glycosylation inhibitor, or thapsigargin, an inhibitor of the sarcoplasmic/endoplasmic reticulum calcium ATPase (SERCA) pump, were observed. Both ER stressors induced a significant decrease in the fluorescence ratio at 8 h, indicating reduction of the ER redox state (Figure 6B) and further reduction of the ER at 16 h (Figure 6C). These results demonstrate that S4 could detect physiological redox changes in the ER as well as changes in the redox state of S4 that are directly affected by addition of oxidoreductants in the ER.

## DISCUSSION

In the present study, we developed an easy-to-use ER redox probe, ERroGFP-iL S4, by introduction of mutations derived from sfGFP. roGFP-iX variants like roGFP-iL, in which an amino-acid residue is inserted next to Cys147, had redox potentials suitable for redox sensing in an oxidative environment such as the ER [15]; however, the inefficient folding of roGFP-iX in the ER hampered the convenient detection of the intracellular redox state using roGFP-iL and roGFP-iE. The four mutations (S30R, Y39N, N105T and I171V) introduced into roGFP-S4 increased the folding efficiency and stability in *E. coli*, as well as the thermostability *in vitro* (Figures 2B and 2C). From the viewpoint of the structural and kinetic analysis of sfGFP, S30R and Y39N greatly improved folding robustness mainly by increasing the electrostatic and hydrogen-bonded stability [24]. I171V caused a local structural change to increase hydrophobic interactions and diminish off-pathway folding routes. N105T improved the stability via the propensity of β-strand formation conferred by introduction of the threonine residue, ultimately increasing expression yield and folding stability [24]. Furthermore, these four mutations extended the dynamic range for glutathione redox change while maintaining *in vitro* redox potentials similar to the original roGFP-iL (Figures 2D and 2E). ERroGFP-S4 was capable of folding efficiently in the ER, which resulted in sufficient fluorescence intensities for monitoring ER redox dynamics. This variant also showed the highest dynamic range among the tested ERroGFP-iL mutants.

ERroGFP-S4 detected the redox change of the ER following a burden to the ER protein quality control system (Figure 6). Overexpression of the Ins2 mutant, which causes accumulation of misfolded proteins, considerably oxidized the ER redox, whereas the wild-type Ins2 caused marginal oxidation of the ER (Figure 6A). The ER oxidation caused by overexpression of proteins folded in the ER appears to be due to an increase in the Ero1L redox cycle following the increased number of folding cycles, since Ero1L generates H_2_O_2_ in every redox cycle by transferring electrons to H_2_O [28,29]. An increased number of futile cycles would cause further oxidation during overexpression of the mutant proteins compared to the wild-type protein. In contrast, drug-induced ER stress reduced the ER redox state 8 h after treatment and caused further reduction at 16 h (Figures 6B and 6C). Reduction of the ER redox state was also observed promptly after tunicamycin and thapsigargin treatment [20,30]. Although calcium depletion-mediated rapid ER reduction was reported to be due to a decrease of PDI mobility in the ER [30], our observed ER reduction following treatment with ER stressors might be due to the UPR including downstream induction of glutathione synthesis [31].

The ER is essential for quality control of secreted and membrane proteins as well as calcium and lipid homoeostasis. Numerous recent reports have demonstrated the association of ER redox with ER homoeostasis [30,32–35]. Our newly generated ERroGFP-S4 is suitable for convenient detection of ER redox change following physiological or pathological changes. Therefore, ERroGFP-S4 will contribute to the identification of unknown mechanisms of redox homoeostasis in the ER, detection of aberrant ER redox states associated with various pathological conditions, and identification of the mechanisms by which aberrant ER redox states are caused.

## Abbreviations

DPS: 4,4’-dipyridyl sulfide
ER: endoplasmic reticulum
Ero1L: Ero1-like protein
PBS (-): phosphate buffered saline without calcium and magnesium
PDI: protein disulfide isomerase
sfGFP: superfolder GFP
UPR: unfolded protein response

## References

[1] Ellgaard L., Helenius A. (2003). Quality control in the endoplasmic reticulum. Nat. Rev. Mol. Cell Biol..

[2] Hetz C. (2012). The unfolded protein response: controlling cell fate decisions under ER stress and beyond. Nat. Rev. Mol. Cell Biol..

[3] Oakes S.A., Papa F.R. (2015). The role of endoplasmic reticulum stress in human pathology. Annu. Rev. Pathol..

[4] Hwang C., Sinskey A.J., Lodish H.F. (1992). Oxidized redox state of glutathione in the endoplasmic reticulum. Science.

[5] Benham A.M. (2012). The protein disulfide isomerase family: key players in health and disease. Antioxid. Redox Signal..

[6] Ushioda R., Hoseki J., Araki K., Jansen G., Thomas D.Y., Nagata K. (2008). ERdj5 is required as a disulfide reductase for degradation of misfolded proteins in the ER. Science.

[7] Hagiwara M., Maegawa K., Suzuki M., Ushioda R., Araki K., Matsumoto Y., Hoseki J., Nagata K., Inaba K. (2011). Structural basis of an ERAD pathway mediated by the ER-resident protein disulfide reductase ERdj5. Mol. Cell.

[8] Ushioda R., Hoseki J., Nagata K. (2013). Glycosylation-independent ERAD pathway serves as a backup system under ER stress. Mol. Biol. Cell.

[9] Schafer F.Q., Buettner G.R. (2001). Redox environment of the cell as viewed through the redox state of the glutathione disulfide/glutathione couple. Free Radic. Biol. Med..

[10] Appenzeller-Herzog C. (2011). Glutathione- and non-glutathione-based oxidant control in the endoplasmic reticulum. J. Cell Sci..

[11] Cuozzo J.W., Kaiser C.A. (1999). Competition between glutathione and protein thiols for disulphide-bond formation. Nat. Cell Biol..

[12] Molteni S.N., Fassio A., Ciriolo M.R., Filomeni G., Pasqualetto E., Fagioli C., Sitia R. (2004). Glutathione limits Ero1-dependent oxidation in the endoplasmic reticulum. J. Biol. Chem..

[13] Jessop C.E., Bulleid N.J. (2004). Glutathione directly reduces an oxidoreductase in the endoplasmic reticulum of mammalian cells. J. Biol. Chem..

[14] Tsunoda S., Avezov E., Zyryanova A., Konno T., Mendes-Silva L., Pinho Melo E., Harding H.P., Ron D. (2014). Intact protein folding in the glutathione-depleted endoplasmic reticulum implicates alternative protein thiol reductants. Elife.

[15] Dixon B.M., Heath S.H., Kim R., Suh J.H., Hagen T.M. (2008). Assessment of endoplasmic reticulum glutathione redox status is confounded by extensive *ex vivo* oxidation. Antioxid. Redox Signal..

[16] Hanson G.T., Aggeler R., Oglesbee D., Cannon M., Capaldi R.A., Tsien R.Y., Remington S.J. (2004). Investigating mitochondrial redox potential with redox-sensitive green fluorescent protein indicators. J. Biol. Chem..

[17] Dooley C.T., Dore T.M., Hanson G.T., Jackson W.C., Remington S.J., Tsien R.Y. (2004). Imaging dynamic redox changes in mammalian cells with green fluorescent protein indicators. J. Biol. Chem..

[18] Merksamer P.I., Trusina A., Papa F.R. (2008). Real-time redox measurements during endoplasmic reticulum stress reveal interlinked protein folding functions. Cell.

[19] Lohman J.R., Remington S.J. (2008). Development of a family of redox-sensitive green fluorescent protein indicators for use in relatively oxidizing subcellular environments. Biochemistry (Mosc).

[20] Birk J., Meyer M., Aller I., Hansen H.G., Odermatt A., Dick T.P., Meyer A.J., Appenzeller-Herzog C. (2013). Endoplasmic reticulum: reduced and oxidized glutathione revisited. J. Cell Sci..

[21] Avezov E., Cross B.C., Kaminski Schierle G.S., Winters M., Harding H.P., Melo E.P., Kaminski C.F., Ron D. (2013). Lifetime imaging of a fluorescent protein sensor reveals surprising stability of ER thiol redox. J. Cell Biol..

[22] van Lith M., Tiwari S., Pediani J., Milligan G., Bulleid N.J. (2011). Real-time monitoring of redox changes in the mammalian endoplasmic reticulum. J. Cell Sci..

[23] Delic M., Mattanovich D., Gasser B. (2010). Monitoring intracellular redox conditions in the endoplasmic reticulum of living yeasts. FEMS Microbiol. Lett..

[24] Pedelacq J.D., Cabantous S., Tran T., Terwilliger T.C., Waldo G.S. (2006). Engineering and characterization of a superfolder green fluorescent protein. Nat. Biotechnol..

[25] Nozaki J., Kubota H., Yoshida H., Naitoh M., Goji J., Yoshinaga T., Mori K., Koizumi A., Nagata K. (2004). The endoplasmic reticulum stress response is stimulated through the continuous activation of transcription factors ATF6 and XBP1 in Ins2+/Akita pancreatic beta cells. Genes Cells.

[26] Yoshinaga T., Nakatome K., Nozaki J.I., Naitoh M., Hoseki J., Kubota H., Nagata K., Koizumi A. (2005). Proinsulin lacking the A7-B7 disulfide bond, Ins2Akita, tends to aggregate due to the exposed hydrophobic surface. Biol. Chem..

[27] Wang J., Takeuchi T., Tanaka S., Kubo S.K., Kayo T., Lu D., Takata K., Koizumi A., Izumi T. (1999). A mutation in the insulin 2 gene induces diabetes with severe pancreatic beta-cell dysfunction in the Mody mouse. J. Clin. Invest..

[28] Gross E., Sevier C.S., Heldman N., Vitu E., Bentzur M., Kaiser C.A., Thorpe C., Fass D. (2006). Generating disulfides enzymatically: reaction products and electron acceptors of the endoplasmic reticulum thiol oxidase Ero1p. Proc. Natl. Acad. Sci. U.S.A..

[29] Wang L., Li S.J., Sidhu A., Zhu L., Liang Y., Freedman R.B., Wang C.C. (2009). Reconstitution of human Ero1-Lalpha/protein-disulfide isomerase oxidative folding pathway *in vitro*. Position-dependent differences in role between the a and a' domains of protein-disulfide isomerase. J. Biol. Chem..

[30] Avezov E., Konno T., Zyryanova A., Chen W., Laine R., Crespillo-Casado A., Melo E.P., Ushioda R., Nagata K., Kaminski C.F. (2015). Retarded PDI diffusion and a reductive shift in poise of the calcium depleted endoplasmic reticulum. BMC Biol.

[31] Harding H.P., Zhang Y., Zeng H., Novoa I., Lu P.D., Calfon M., Sadri N., Yun C., Popko B., Paules R. (2003). An integrated stress response regulates amino acid metabolism and resistance to oxidative stress. Mol. Cell.

[32] Hisatsune C., Ebisui E., Usui M., Ogawa N., Suzuki A., Mataga N., Takahashi-Iwanaga H., Mikoshiba K. (2015). ERp44 exerts redox-dependent control of blood pressure at the ER. Mol. Cell.

[33] Puigpinos J., Casas C., Herrero E. (2015). Altered intracellular calcium homeostasis and endoplasmic reticulum redox state in Saccharomyces cerevisiae cells lacking Grx6 glutaredoxin. Mol. Biol. Cell.

[34] Higa A., Taouji S., Lhomond S., Jensen D., Fernandez-Zapico M.E., Simpson J.C., Pasquet J.M., Schekman R., Chevet E. (2014). Endoplasmic reticulum stress-activated transcription factor ATF6alpha requires the disulfide isomerase PDIA5 to modulate chemoresistance. Mol. Cell. Biol..

[35] Eletto D., Dersh D., Gidalevitz T., Argon Y. (2014). Protein disulfide isomerase A6 controls the decay of IRE1alpha signaling via disulfide-dependent association. Mol. Cell.

